# Supplementary material for: Alcohol use disorder and body mass index show genetic pleiotropy and shared neural associations
Source: medRxiv. 2024 Dec 14:2024.05.03.24306773. Originally published 2024 May 5. Preprint. [Version 2] doi: 10.1101/2024.05.03.24306773 (PMC11092735; doi:10.1101/2024.05.03.24306773)

medRxiv preprint doi: https://doi.org/10.1101/2024.05.03.24306773; this version posted December 14, 2024. The copyright holder for this preprint (which was not certified by peer review) is the author/funder, who has granted medRxiv a license to display the preprint in perpetuity. This article is a US Government work. It is not subject to copyright under 17 USC 105 and is also made available for use under a CC0 license.

Figure S1. Conditional Q-Q plots showing the distribution of observed versus expected $-\log_{10}$ *p*-values for the primary phenotypes (AUD and BMI) for SNPs conditional on associations of a secondary trait at three p-value strata ($p \leq 0.1$, 0.01, and 0.001). ADHD = attention deficit hyperactivity disorder, AUD = alcohol use disorder, BMI = body mass index, MDD = major depressive disorder, SCZ = schizophrenia.

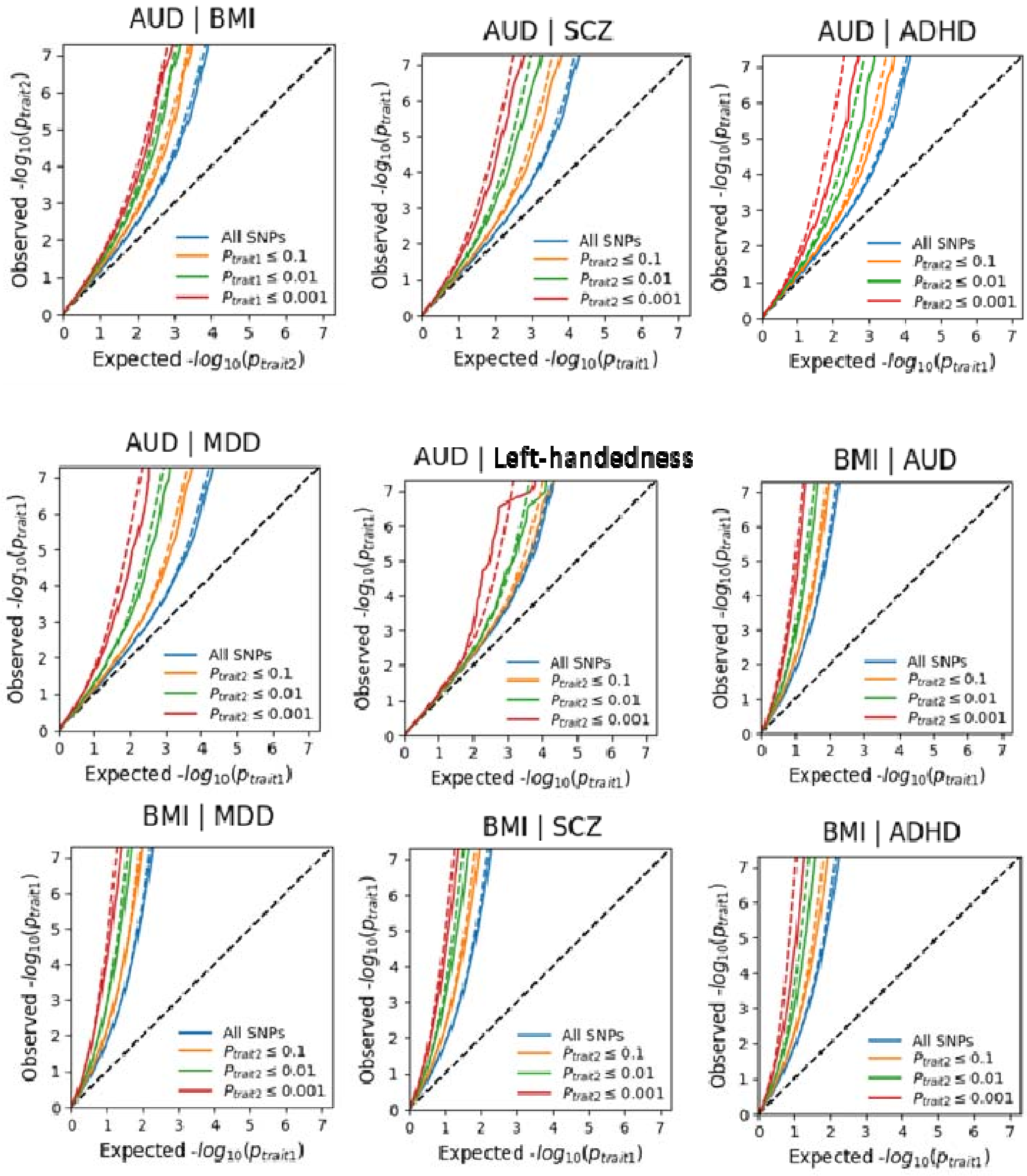

medRxiv preprint doi: https://doi.org/10.1101/2024.05.03.24306773; this version posted December 14, 2024. The copyright holder for this preprint (which was not certified by peer review) is the author/funder, who has granted medRxiv a license to display the preprint in perpetuity.
This article is a US Government work. It is not subject to copyright under 17 USC 105 and is also made available for use under a CC0 license.

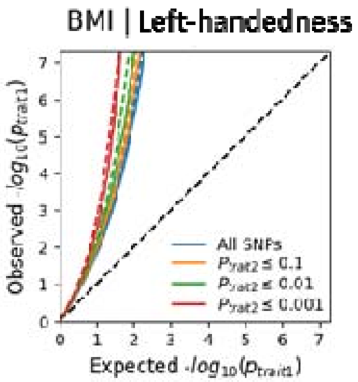

medRxiv preprint doi: https://doi.org/10.1101/2024.05.03.24306773; this version posted December 14, 2024. The copyright holder for this preprint (which was not certified by peer review) is the author/funder, who has granted medRxiv a license to display the preprint in perpetuity.
This article is a US Government work. It is not subject to copyright under 17 USC 105 and is also made available for use under a CC0 license.

Figure S2. **A)** Differential gene expression (DEG) in 30 general GTEx tissues for genes linked to lead SNPs in distinct loci significantly associated with both AUD and BMI. Significant enrichment (p<.05 after Bonferroni correction) is highlighted in red. **B)** Gene expression heatmap for 30 GTEx general tissues for genes linked to lead SNPs in distinct loci significantly associated with both AUD and BMI.

A

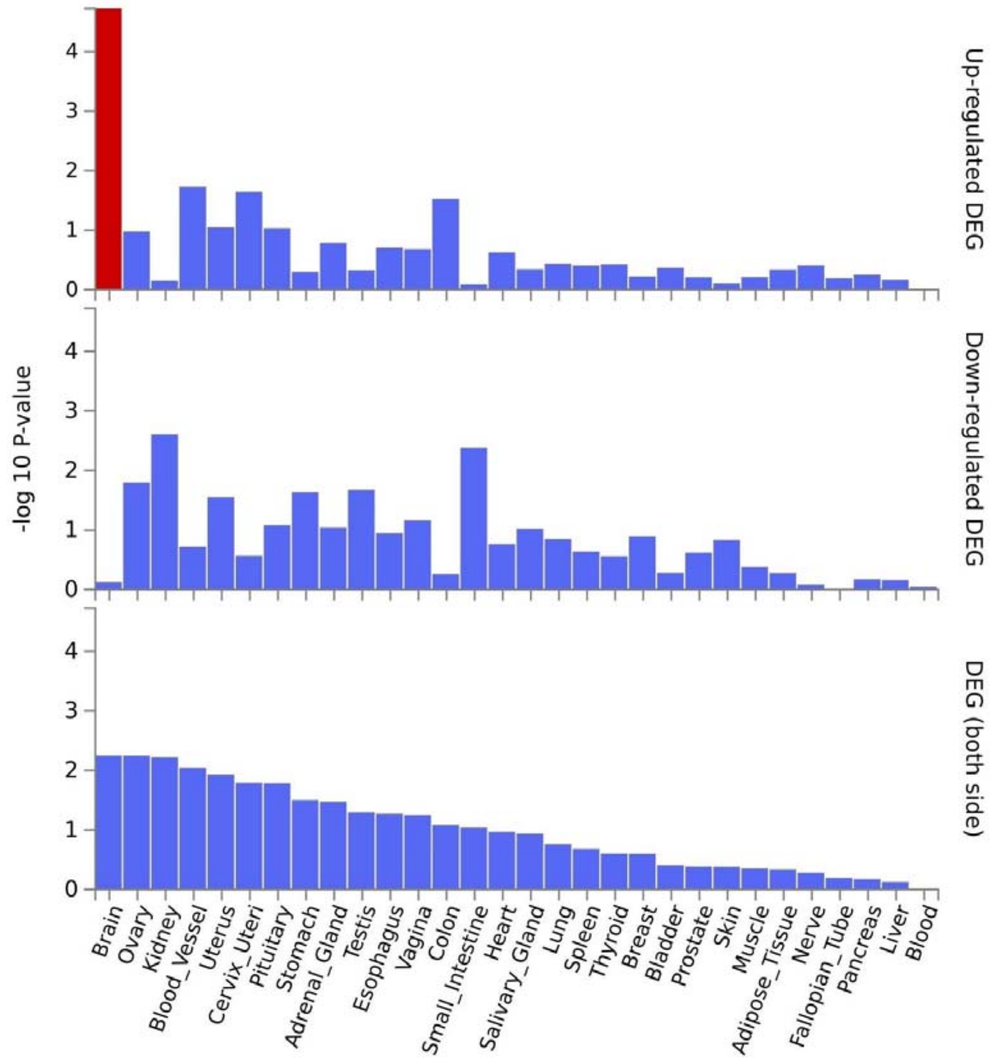

medRxiv preprint doi: https://doi.org/10.1101/2024.05.03.24306773; this version posted December 14, 2024. The copyright holder for this preprint (which was not certified by peer review) is the author/funder, who has granted medRxiv a license to display the preprint in perpetuity. This article is a US Government work. It is not subject to copyright under 17 USC 105 and is also made available for use under a CC0 license.

B

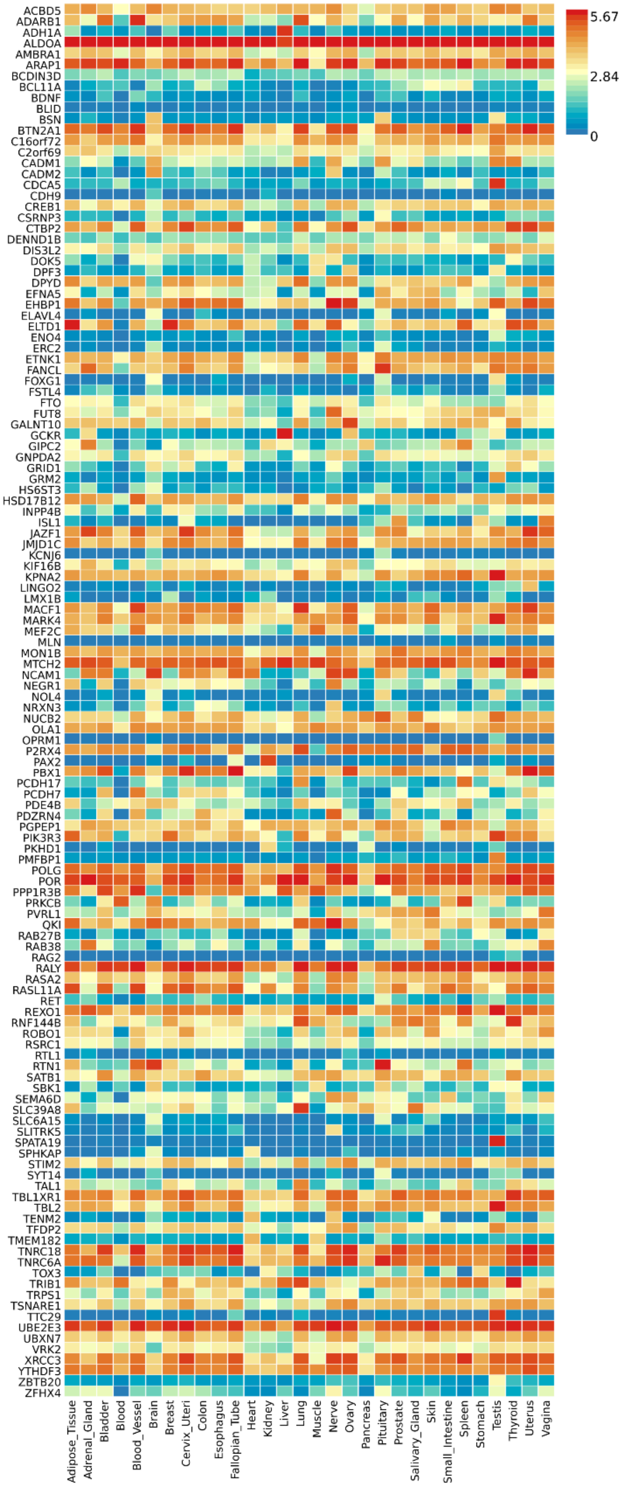

medRxiv preprint doi: https://doi.org/10.1101/2024.05.03.24306773; this version posted December 14, 2024. The copyright holder for this preprint (which was not certified by peer review) is the author/funder, who has granted medRxiv a license to display the preprint in perpetuity. This article is a US Government work. It is not subject to copyright under 17 USC 105 and is also made available for use under a CC0 license.

Figure S3. Gene expression heatmap for 54 GTEx tissues for genes linked to lead SNPs in distinct loci significantly associated with both AUD and BMI.

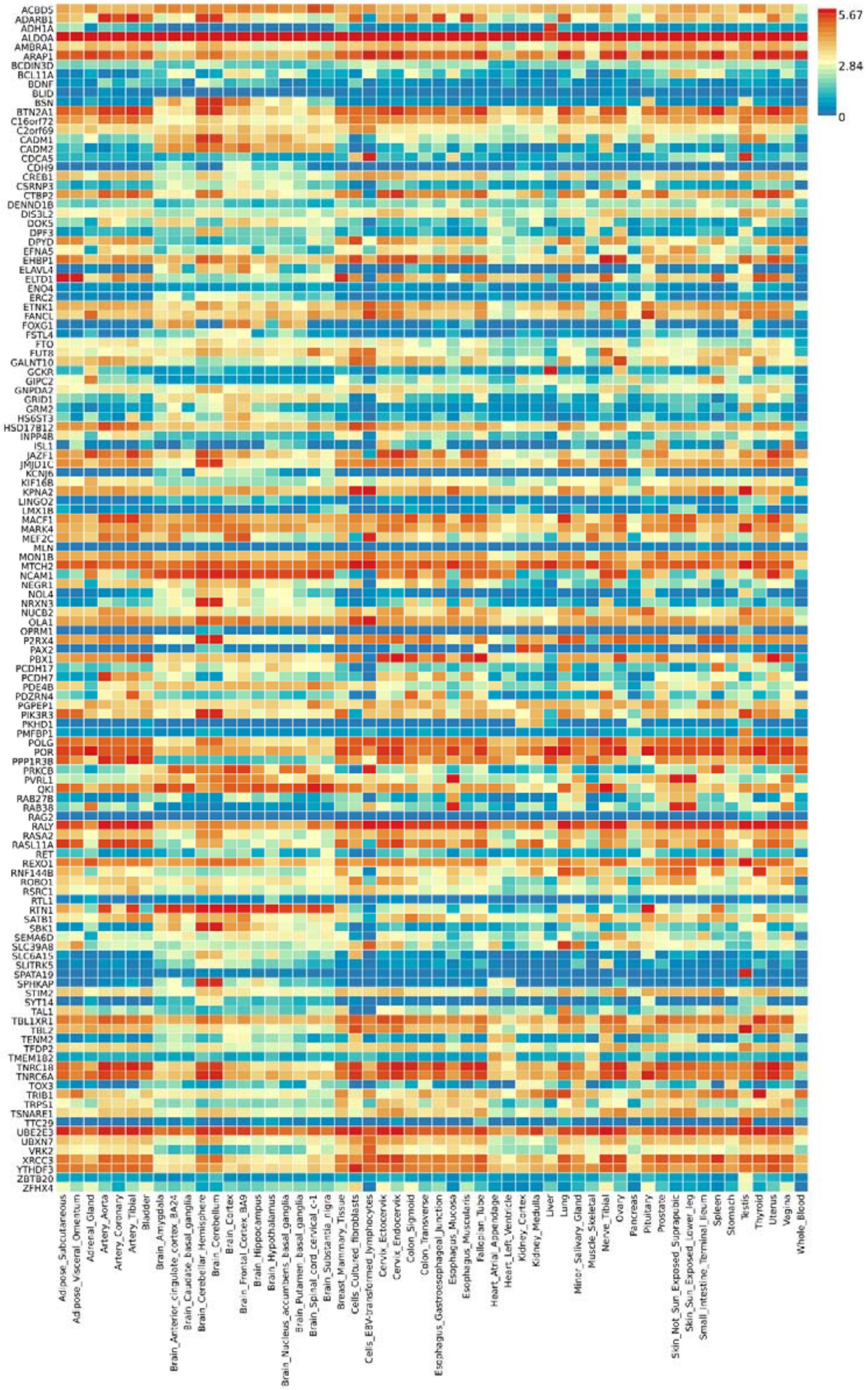

medRxiv preprint doi: https://doi.org/10.1101/2024.05.03.24306773; this version posted December 14, 2024. The copyright holder for this preprint (which was not certified by peer review) is the author/funder, who has granted medRxiv a license to display the preprint in perpetuity. This article is a US Government work. It is not subject to copyright under 17 USC 105 and is also made available for use under a CC0 license.

Figure S4. Concordant enrichment. **A)** Differential gene expression (DEG) in 30 general GTEx tissues for genes linked to concordant lead SNPs in distinct loci significantly associated with both AUD and BMI. There was no significant enrichment (p<.05 after Bonferroni correction). **B)** Gene expression heatmap for 30 GTEx general tissues for genes linked to concordant lead SNPs in distinct loci significantly associated with both AUD and BMI. **C)** Differential gene expression (DEG) in 54 general GTEx tissues for genes linked to concordant lead SNPs in distinct loci significantly associated with both AUD and BMI. Significant enrichment (p<.05 after Bonferroni correction) is highlighted in red. **D)** Gene expression heatmap for 54 GTEx general tissues for genes linked to concordant lead SNPs in distinct loci significantly associated with both AUD and BMI.

medRxiv preprint doi: https://doi.org/10.1101/2024.05.03.24306773; this version posted December 14, 2024. The copyright holder for this preprint (which was not certified by peer review) is the author/funder, who has granted medRxiv a license to display the preprint in perpetuity.
This article is a US Government work. It is not subject to copyright under 17 USC 105 and is also made available for use under a CC0 license.

A

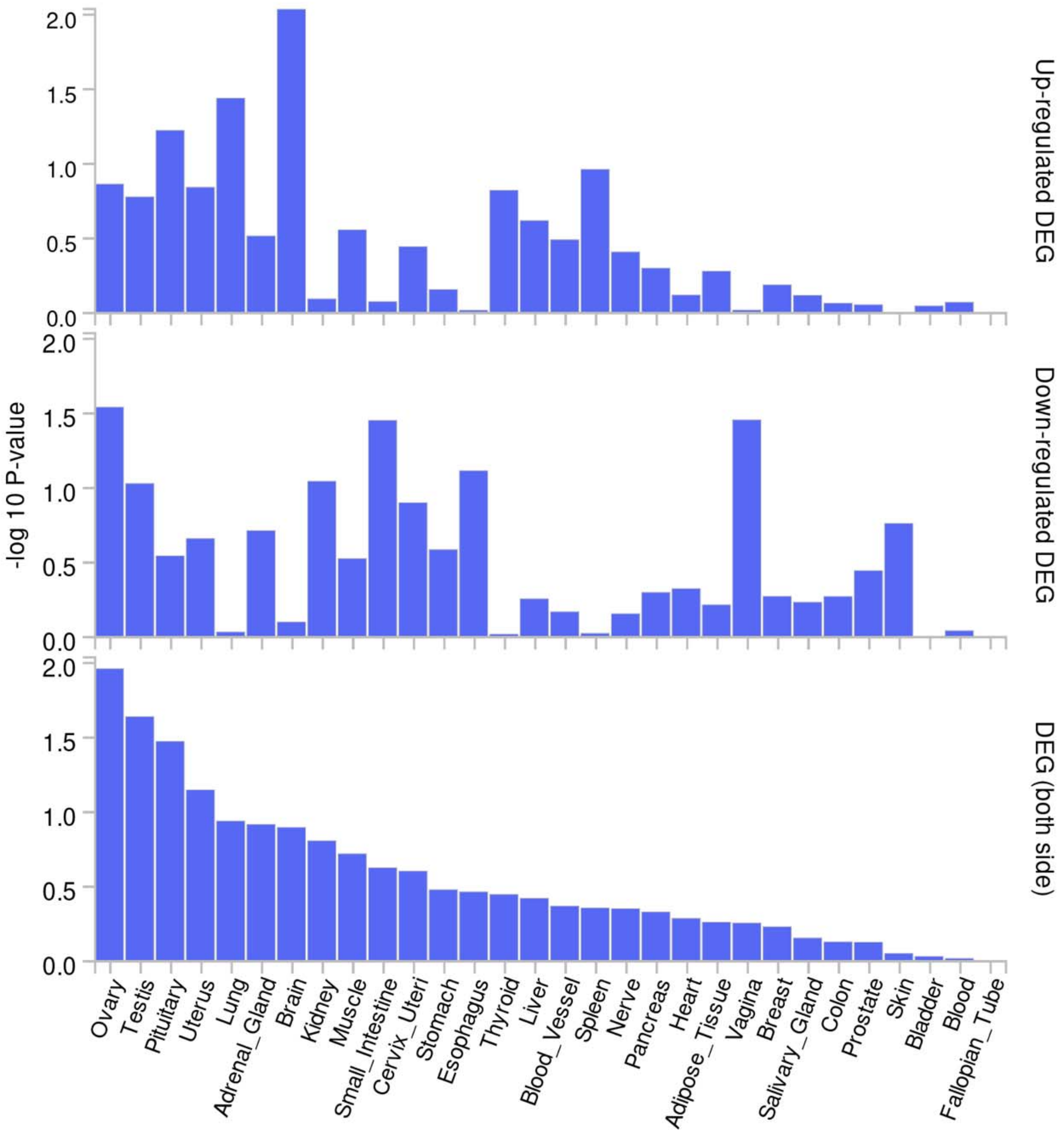

medRxiv preprint doi: https://doi.org/10.1101/2024.05.03.24306773; this version posted December 14, 2024. The copyright holder for this preprint (which was not certified by peer review) is the author/funder, who has granted medRxiv a license to display the preprint in perpetuity.
This article is a US Government work. It is not subject to copyright under 17 USC 105 and is also made available for use under a CC0 license.

B

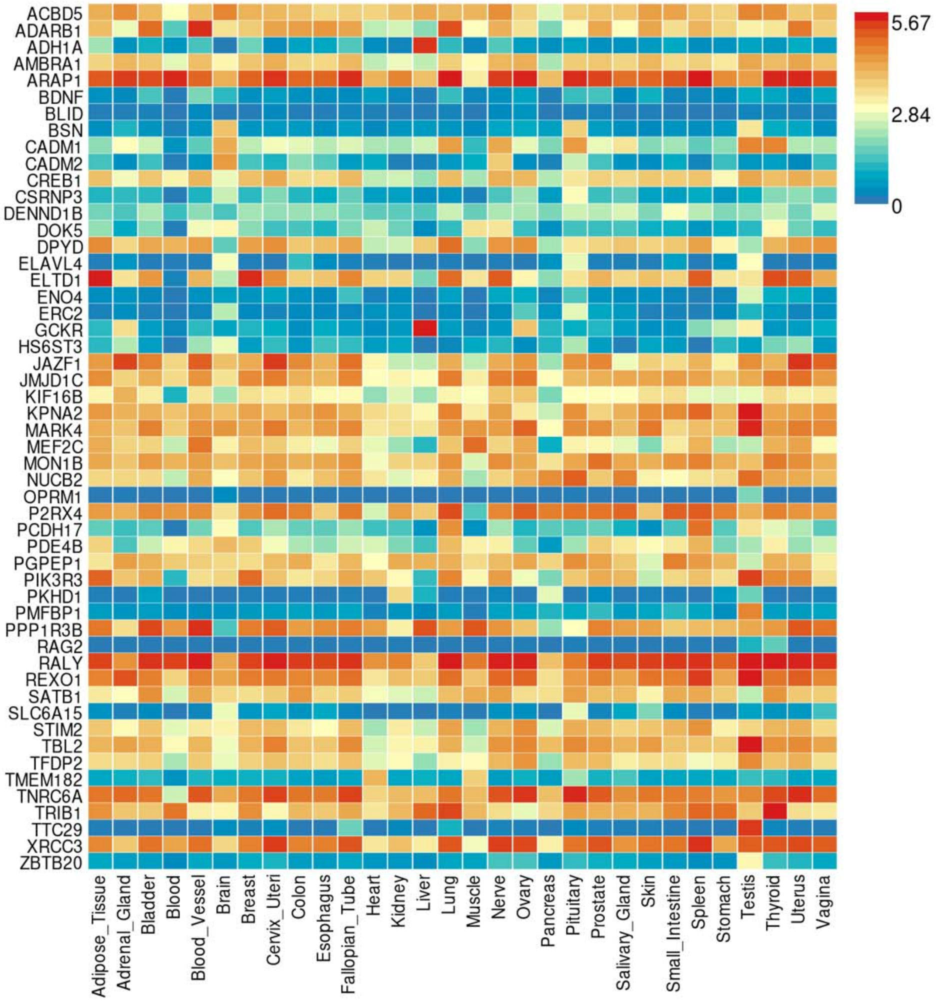

medRxiv preprint doi: https://doi.org/10.1101/2024.05.03.24306773; this version posted December 14, 2024. The copyright holder for this preprint (which was not certified by peer review) is the author/funder, who has granted medRxiv a license to display the preprint in perpetuity. This article is a US Government work. It is not subject to copyright under 17 USC 105 and is also made available for use under a CC0 license.

C

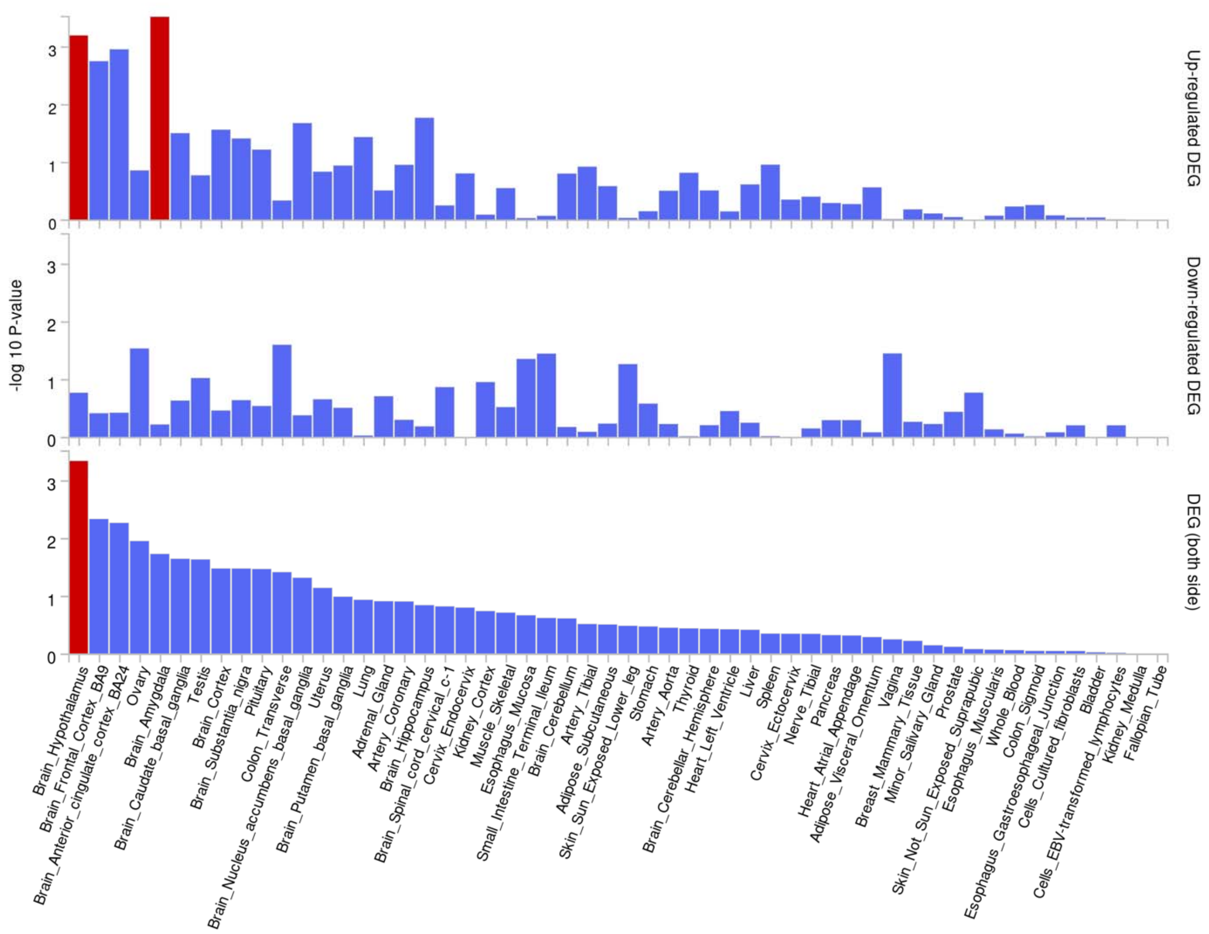

medRxiv preprint doi: https://doi.org/10.1101/2024.05.03.24306773; this version posted December 14, 2024. The copyright holder for this preprint (which was not certified by peer review) is the author/funder, who has granted medRxiv a license to display the preprint in perpetuity. This article is a US Government work. It is not subject to copyright under 17 USC 105 and is also made available for use under a CC0 license.

D

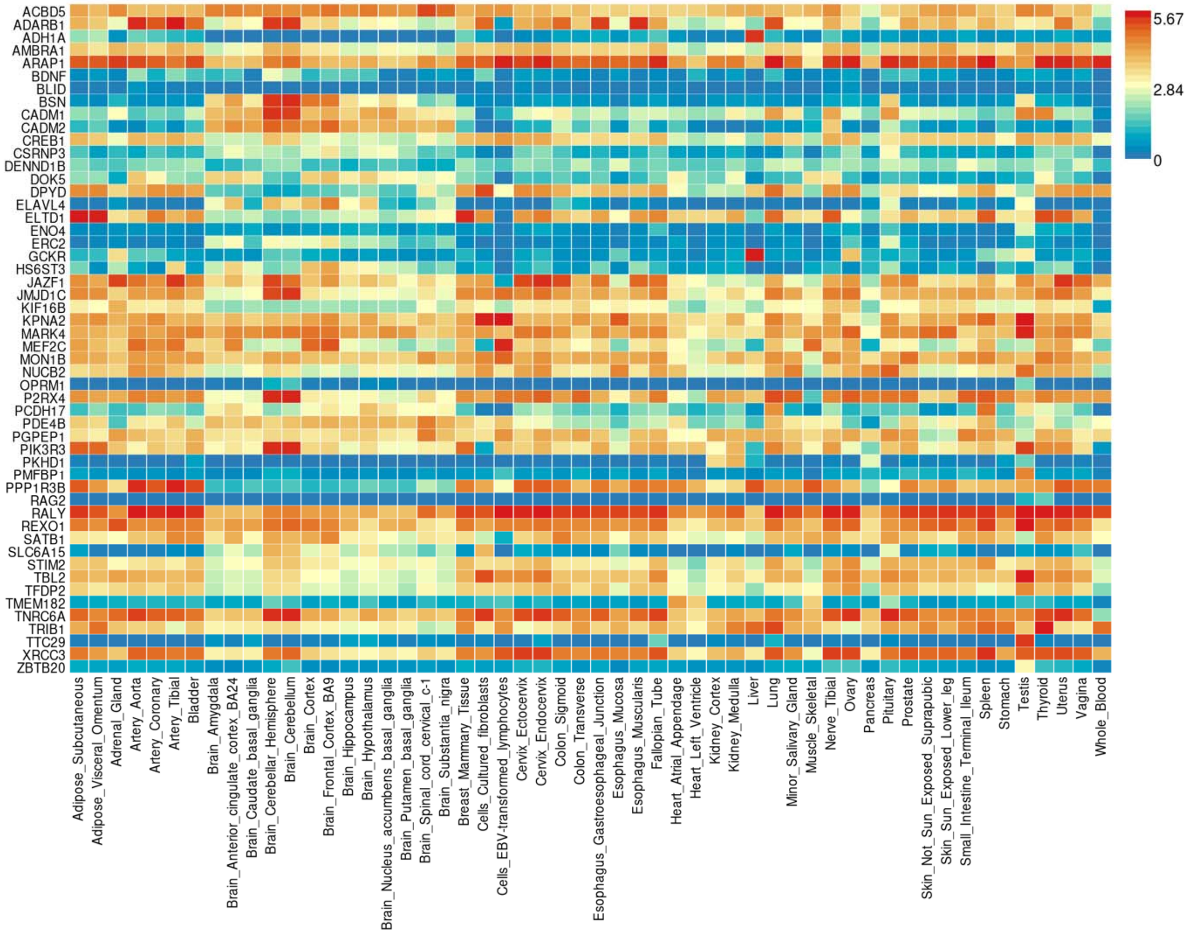

medRxiv preprint doi: https://doi.org/10.1101/2024.05.03.24306773; this version posted December 14, 2024. The copyright holder for this preprint (which was not certified by peer review) is the author/funder, who has granted medRxiv a license to display the preprint in perpetuity. This article is a US Government work. It is not subject to copyright under 17 USC 105 and is also made available for use under a CC0 license.

Figure S5. Discordant enrichment. **A)** Differential gene expression (DEG) in 30 general GTEx tissues for genes linked to discordant lead SNPs in distinct loci significantly associated with both AUD and BMI. There was no significant enrichment (p<.05 after Bonferroni correction). **B)** Gene expression heatmap for 30 GTEx general tissues for genes linked to discordant lead SNPs in distinct loci significantly associated with both AUD and BMI. **C)** Differential gene expression (DEG) in 54 general GTEx tissues for genes linked to discordant lead SNPs in distinct loci significantly associated with both AUD and BMI. Significant enrichment (p<.05 after Bonferroni correction) is highlighted in red. **D)** Gene expression heatmap for 54 GTEx general tissues for genes linked to discordant lead SNPs in distinct loci significantly associated with both AUD and BMI.

medRxiv preprint doi: https://doi.org/10.1101/2024.05.03.24306773; this version posted December 14, 2024. The copyright holder for this preprint (which was not certified by peer review) is the author/funder, who has granted medRxiv a license to display the preprint in perpetuity. This article is a US Government work. It is not subject to copyright under 17 USC 105 and is also made available for use under a CC0 license.

A

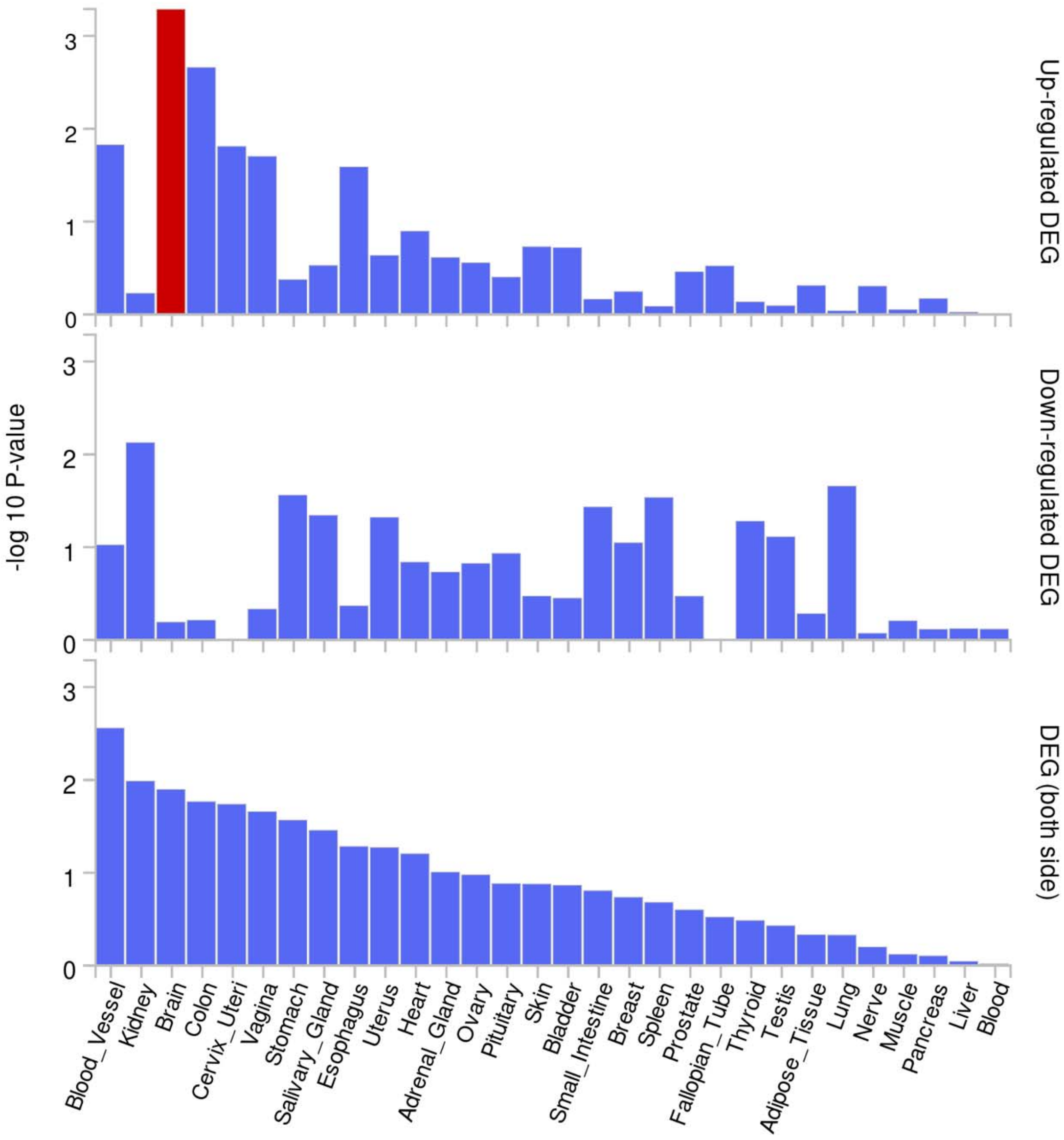

medRxiv preprint doi: https://doi.org/10.1101/2024.05.03.24306773; this version posted December 14, 2024. The copyright holder for this preprint (which was not certified by peer review) is the author/funder, who has granted medRxiv a license to display the preprint in perpetuity.
This article is a US Government work. It is not subject to copyright under 17 USC 105 and is also made available for use under a CC0 license.

B

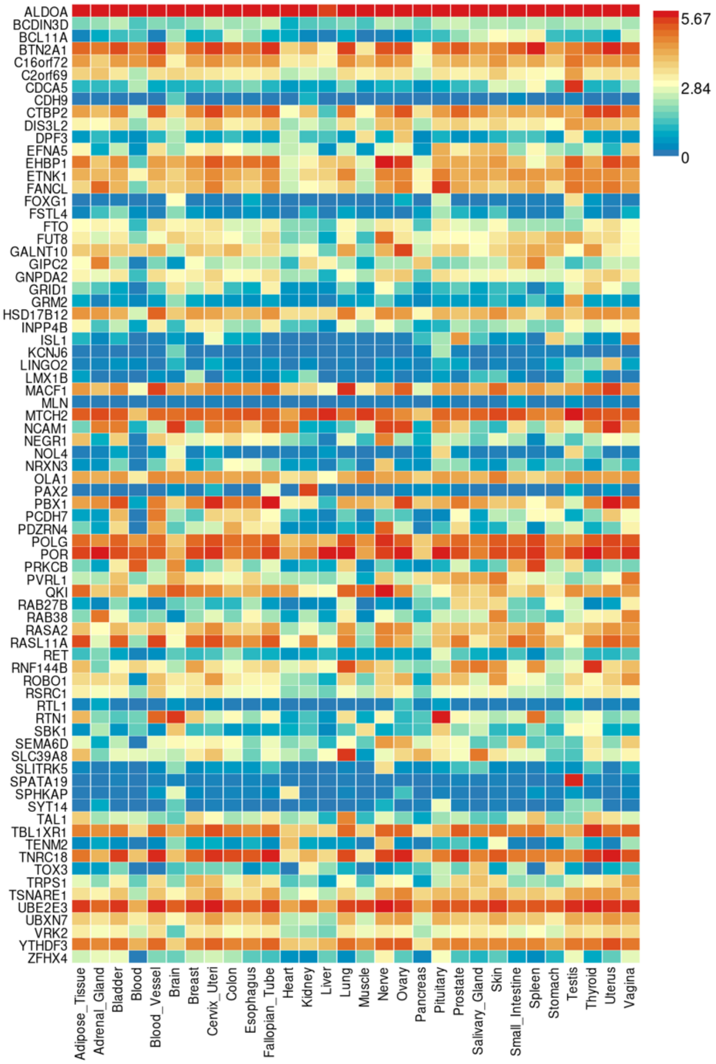

medRxiv preprint doi: https://doi.org/10.1101/2024.05.03.24306773; this version posted December 14, 2024. The copyright holder for this preprint (which was not certified by peer review) is the author/funder, who has granted medRxiv a license to display the preprint in perpetuity. This article is a US Government work. It is not subject to copyright under 17 USC 105 and is also made available for use under a CC0 license.

C

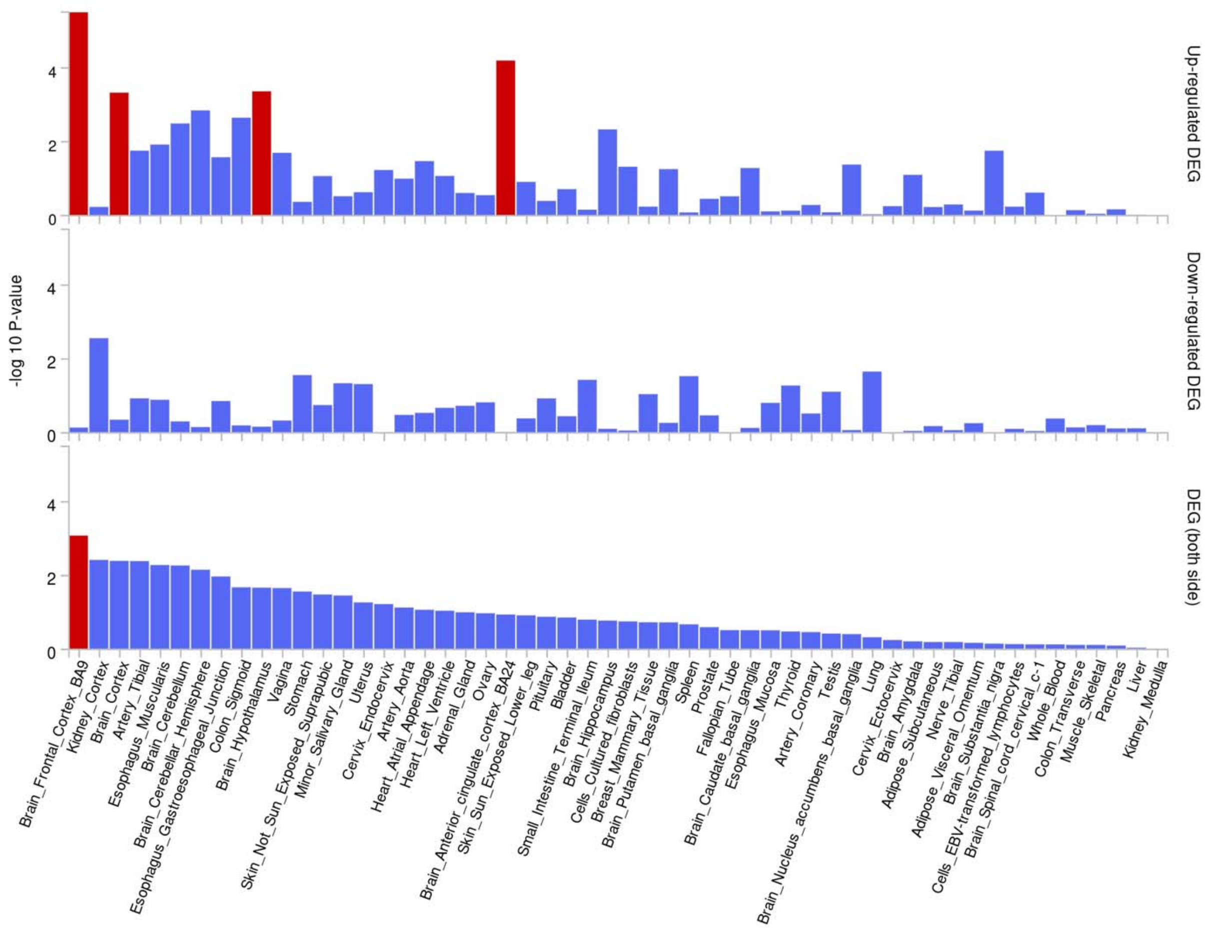

medRxiv preprint doi: https://doi.org/10.1101/2024.05.03.24306773; this version posted December 14, 2024. The copyright holder for this preprint (which was not certified by peer review) is the author/funder, who has granted medRxiv a license to display the preprint in perpetuity. This article is a US Government work. It is not subject to copyright under 17 USC 105 and is also made available for use under a CC0 license.

D

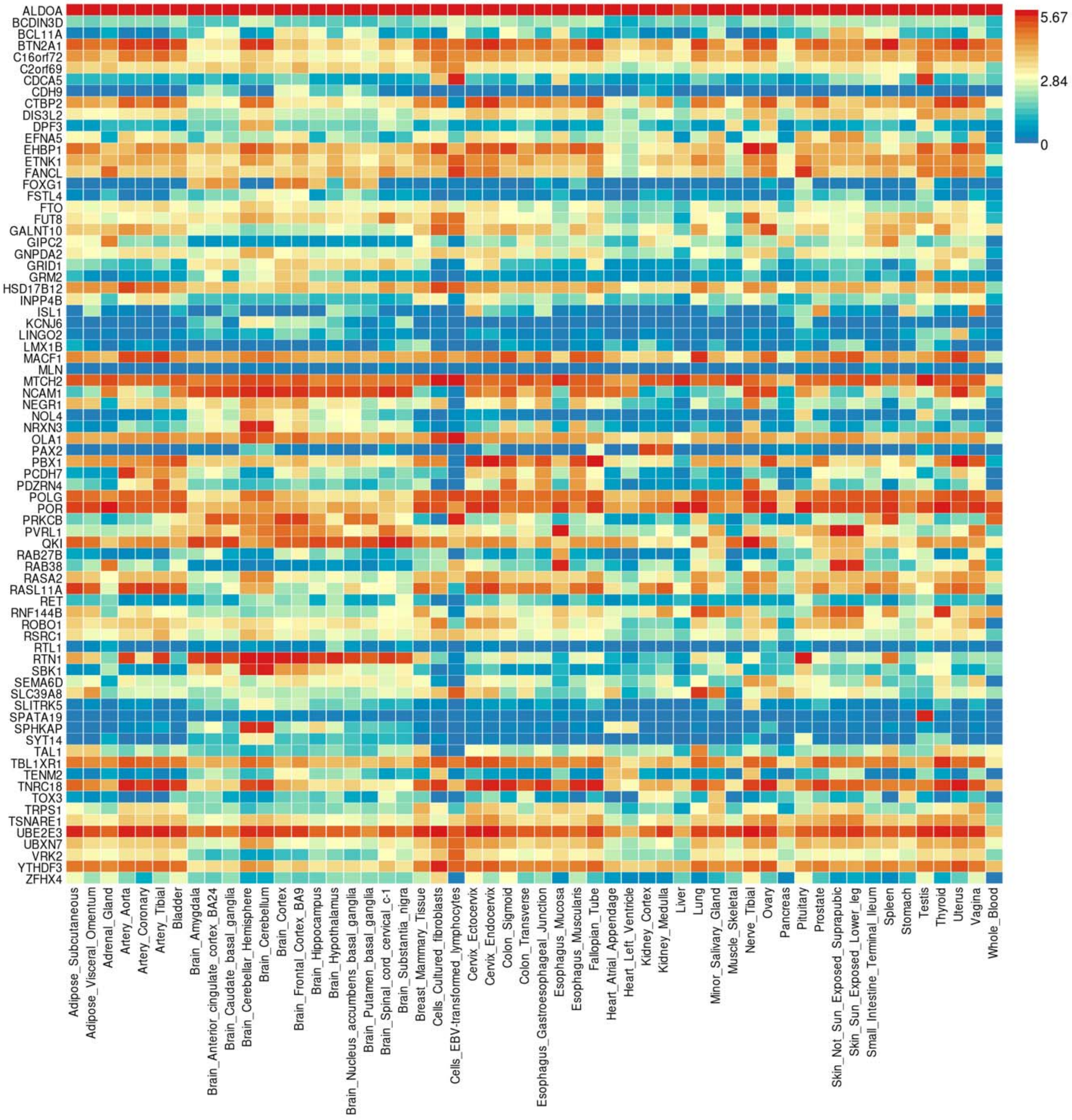

medRxiv preprint doi: https://doi.org/10.1101/2024.05.03.24306773; this version posted December 14, 2024. The copyright holder for this preprint (which was not certified by peer review) is the author/funder, who has granted medRxiv a license to display the preprint in perpetuity. This article is a US Government work. It is not subject to copyright under 17 USC 105 and is also made available for use under a CC0 license.

Figure S6. Structural brain features associated with AUD risk. LD-adjusted Z-scores are provided for region-specific associations of structural IDPs with AUD risk.

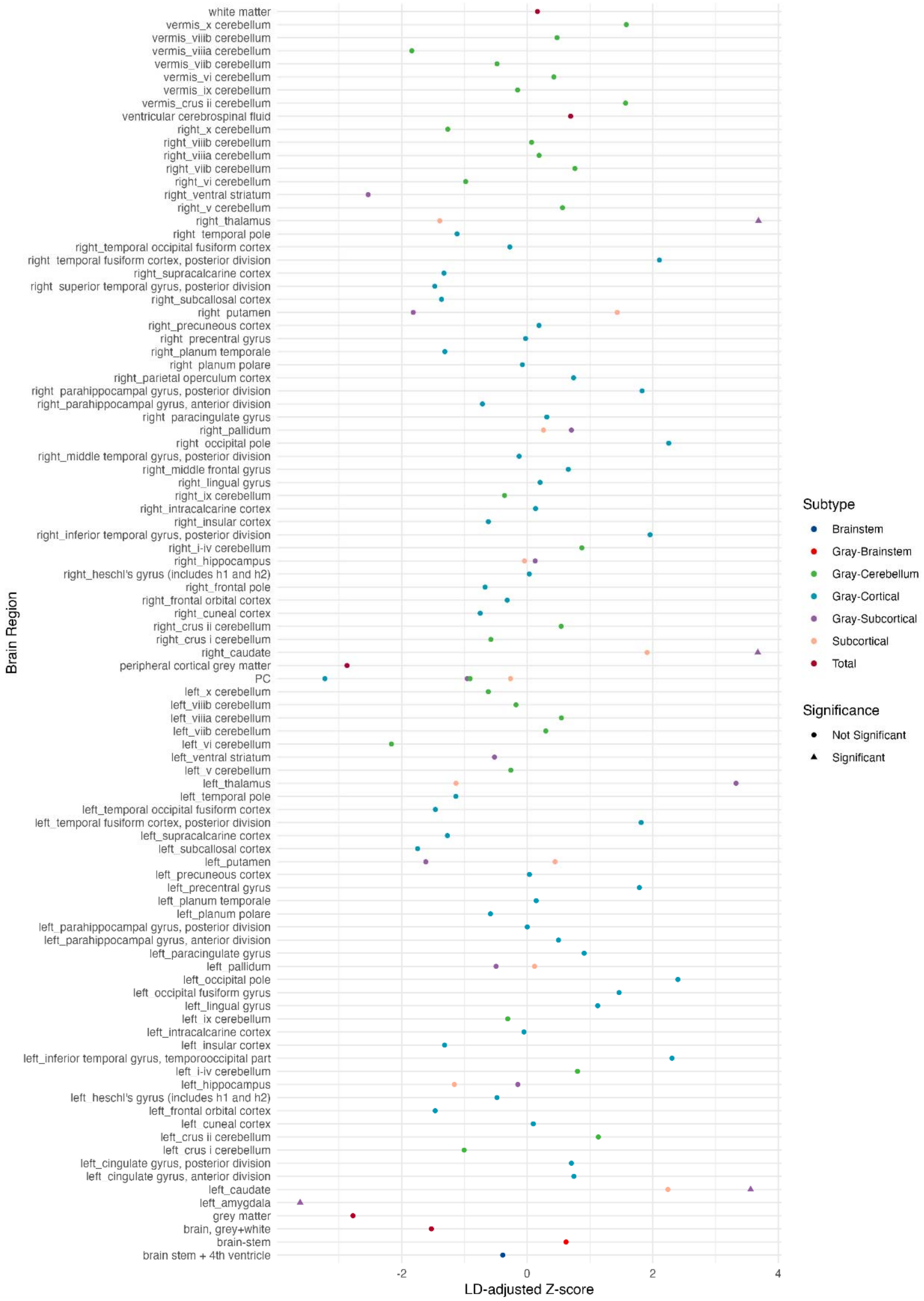

medRxiv preprint doi: https://doi.org/10.1101/2024.05.03.24306773; this version posted December 14, 2024. The copyright holder for this preprint (which was not certified by peer review) is the author/funder, who has granted medRxiv a license to display the preprint in perpetuity. This article is a US Government work. It is not subject to copyright under 17 USC 105 and is also made available for use under a CC0 license.

Figure S7. Diffusion features associated with AUD risk. LD-adjusted Z-scores are provided for region-specific associations of diffusion MRI IDPs with AUD risk.

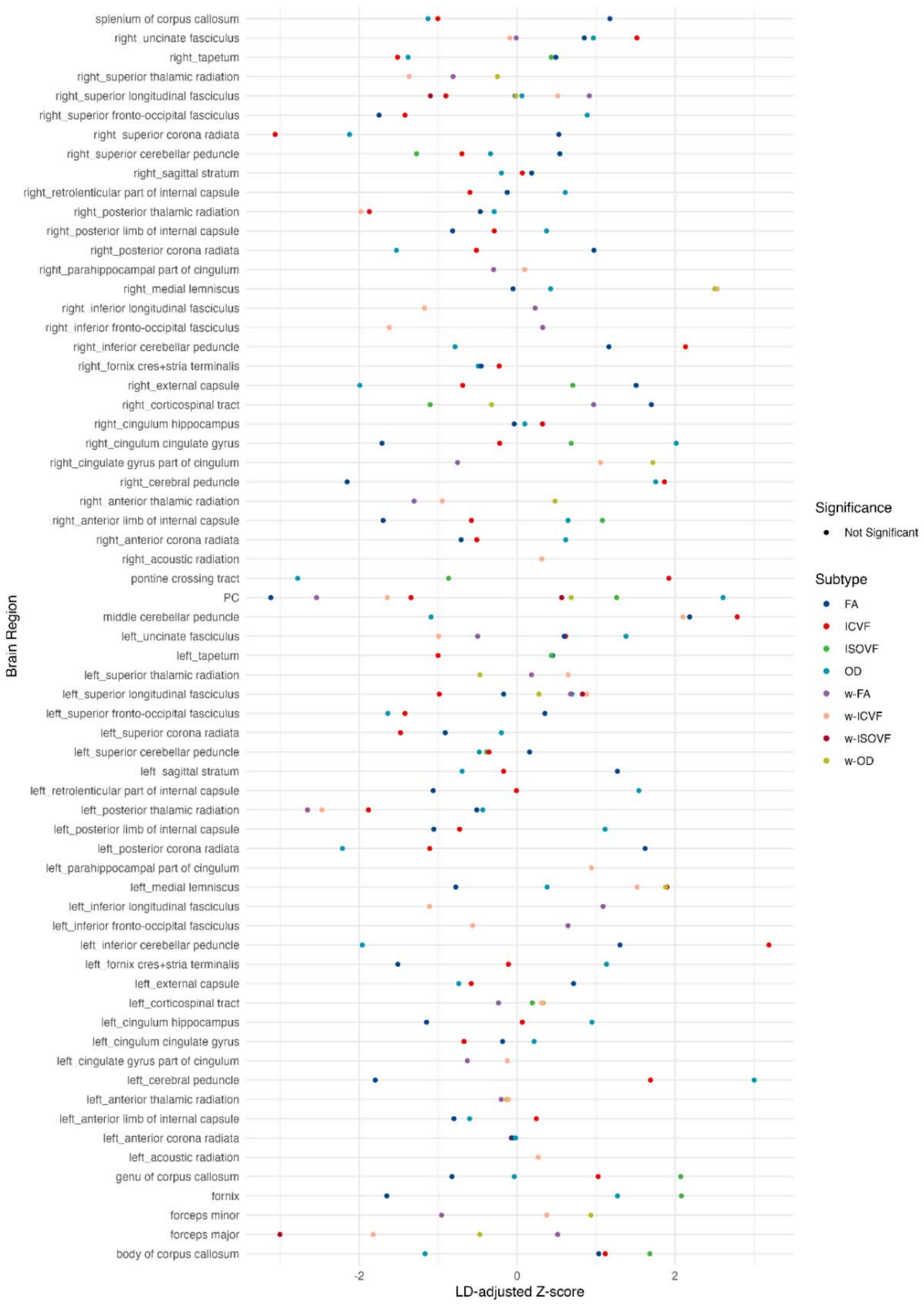

medRxiv preprint doi: https://doi.org/10.1101/2024.05.03.24306773; this version posted December 14, 2024. The copyright holder for this preprint (which was not certified by peer review) is the author/funder, who has granted medRxiv a license to display the preprint in perpetuity. This article is a US Government work. It is not subject to copyright under 17 USC 105 and is also made available for use under a CC0 license.

Figure S8. Structural features associated with BMI risk. LD-adjusted Z-scores are provided for region-specific associations of structural IDPs with BMI risk.

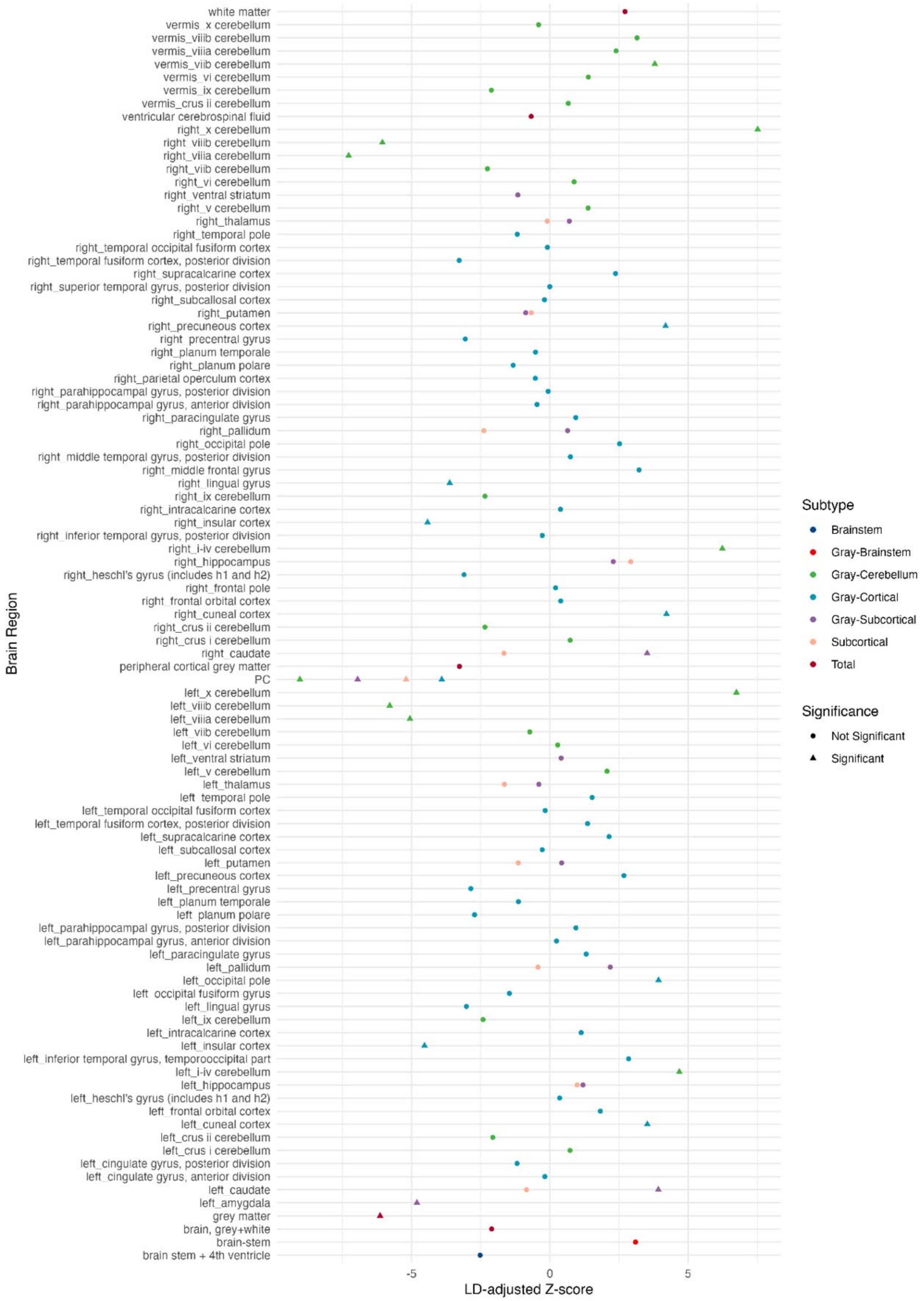

medRxiv preprint doi: https://doi.org/10.1101/2024.05.03.24306773; this version posted December 14, 2024. The copyright holder for this preprint (which was not certified by peer review) is the author/funder, who has granted medRxiv a license to display the preprint in perpetuity. This article is a US Government work. It is not subject to copyright under 17 USC 105 and is also made available for use under a CC0 license.

Figure S9. Diffusion features associated with BMI risk. LD-adjusted Z-scores are provided for region-specific associations of diffusion MRI IDPs with BMI risk.

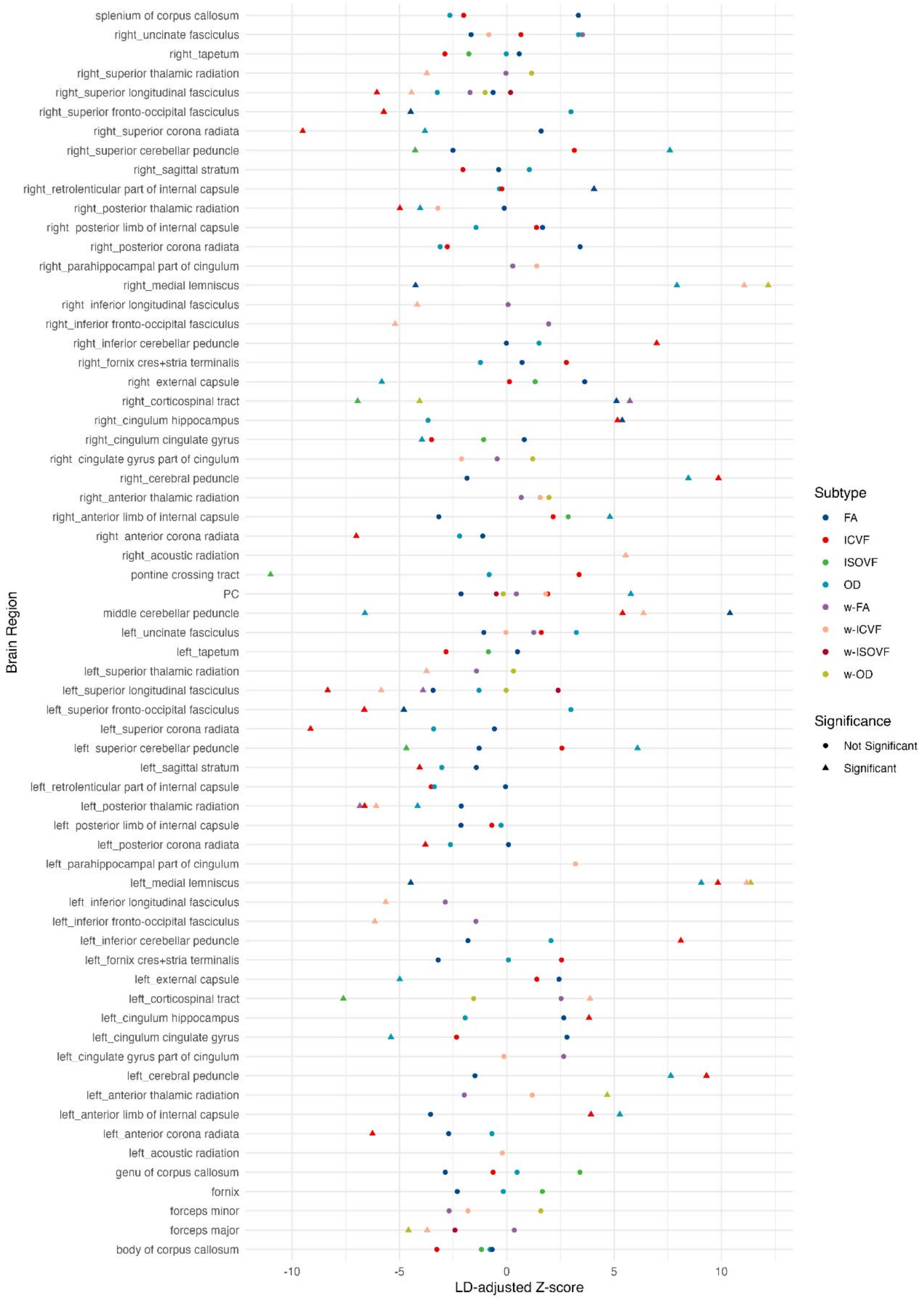

Supplement: 1 [file NIHPP2024.05.03.24306773V2-supplement-1.pdf]
